# Metabolomic Biomarkers Are Associated With Area of the Pons in Fragile X Premutation Carriers at Risk for Developing FXTAS

**DOI:** 10.3389/fpsyt.2021.691717

**Published:** 2021-08-16

**Authors:** Marwa Zafarullah, Blythe Durbin-Johnson, Emily S. Fourie, David R. Hessl, Susan M. Rivera, Flora Tassone

**Affiliations:** ^1^Department of Biochemistry and Molecular Medicine, University of California, Davis, School of Medicine, Sacramento, CA, United States; ^2^Division of Biostatistics, School of Medicine, University of California, Davis, Davis, CA, United States; ^3^Center for Mind and Brain, University of California, Davis, Davis, CA, United States; ^4^Department of Psychology, University of California, Davis, Davis, CA, United States; ^5^MIND Institute, University of California, Davis Medical Center, Sacramento, CA, United States; ^6^Department of Psychiatry and Behavioral Sciences, University of California, Davis Medical Center, Sacramento, CA, United States

**Keywords:** fragile X-associated tremor/ataxia syndrome, area of the pons, metabolic biomarkers, brain measures, lipids, premutation carriers

## Abstract

Fragile X-associated tremor/ataxia syndrome (FXTAS) is a late adult-onset neurodegenerative disorder that affects movement and cognition in male and female carriers of a premutation allele (55–200 CGG repeats; PM) in the fragile X mental retardation (*FMR1*) gene. It is currently unknown how the observed brain changes are associated with metabolic signatures in individuals who develop the disorder over time. The primary objective of this study was to investigate the correlation between longitudinal changes in the brain (area of the pons, midbrain, and MCP width) and the changes in the expression level of metabolic biomarkers of early diagnosis and progression of FXTAS in PM who, as part of an ongoing longitudinal study, emerged into two distinct categories. These included those who developed symptoms of FXTAS (converters, CON) at subsequent visits and those who did not meet the criteria of diagnosis (non-converters, NCON) and were compared to age-matched healthy controls (HC). We assessed CGG repeat allele size by Southern Blot and PCR analysis. Magnetic Resonance Imaging (MRIs) acquisition was obtained on a 3T Siemens Trio scanner and metabolomic profile was obtained by ultra-performance liquid chromatography, accurate mass spectrometer, and an Orbitrap mass analyzer. Our findings indicate that differential metabolite levels are linked with the area of the pons between healthy control and premutation groups. More specifically, we observed a significant association of ceramides and mannonate metabolites with a decreased area of the pons, both at visit 1 (V1) and visit 2 (V2) only in the CON as compared to the NCON group suggesting their potential role in the development of the disorder. In addition, we found a significant correlation of these metabolic signatures with the FXTAS stage at V2 indicating their contribution to the progression and pathogenesis of FXTAS. Interestingly, these metabolites, as part of lipid and sphingolipid lipids pathways, provide evidence of the role that their dysregulation plays in the development of FXTAS and inform us as potential targets for personalized therapeutic development.

## Introduction

Aging is a complex and evolutionarily conserved process that is found to be one of the main risk factors for a number of human neurodegenerative disorders (1). Aging and many aging-associated disorders share a range of molecular or cellular pathologies, which can involve a dysregulated energy balance. Increasing evidence suggests that metabolic alterations can strongly influence the development and the progression of various neurodegenerative disorders. Although the brain represents only 2% of the total body weight, it accounts for 20% of an individual's energy expenditure at rest (2). Thus, compromised energy metabolism and adverse changes, are potentially contributing to increased vulnerability of the brain to develop neurodevelopmental and neurodegenerative processes (3).

Fragile X-associated Tremor/Ataxia Syndrome (FXTAS) is a late-onset neurodegenerative disorder, mostly affecting carriers of the fragile X mental retardation 1 (*FMR1*) gene mutation after the age of 50. Currently, there is no effective treatment for FXTAS, and the cognitive and/or motor symptoms progressively worsen over time, causing reduced quality of life, increased medical costs, and eventually, death. FXTAS is caused by the expanded CGG repeats (55–200 CGG) within the 5′UTR of the *FMR1* gene. In normal healthy individuals, the number of CGG repeats lies between 5 and 54 while individuals carrying alleles with a CGG repeat expansion >200 develop fragile X syndrome (FXS), the most common form of intellectual disability and known monogenic cause of autism spectrum disorders (ASD) (4). The high prevalence of the premutation allele among the general population (1:110–200 females and 1:430 males), leads to an estimate of approximately 1.5 million individuals in the general US population being at risk for *FMR1* associated disorders, over their life spans. In addition, among the PM population, an estimated 40–75% of male and 8–16% of female PMs are at risk of developing FXTAS (5, 6).

FXTAS core features include progressive intention tremor and cerebellar gait ataxia, autonomic dysfunction, and parkinsonism. Neuropathologically, it is characterized by the presence of ubiquitin-positive intranuclear inclusions in neurons and astrocytes throughout the brain and in Purkinje cells (7). In addition to the clinical and neuropathological features, the radiological signs, including white matter hyperintensities (wmhs) in the middle cerebellar peduncles (the “MCP sign”) (8) also contribute to the diagnosis of FXTAS. Similarly, a significant prevalence of wmhs in the splenium of the corpus callosum (9, 10), generalized brain atrophy, increased T2 signal in area of the pons and periventricular regions along with the subcortical gray matter damage with atrophy of the midbrain, are part of the pathogenesis of FXTAS (5, 11).

The brainstem is the central axis of the brain and both of its regions, the area of the pons and the midbrain, play an important role in sensation and movement (12). The upper area of the pons and midbrain tegmentum are the main components of the ascending reticular activating system and associated with various other neurodegenerative disorders (13). Measurements of these areas have been shown previously to successfully differentiate subcortical movement disorders, such as Parkinson's disease (14), which presents with resting tremor that has also been observed in FXTAS. In addition, middle cerebellar peduncle (MCP) width showed a great sensitivity and specificity in differentiating multiple system atrophy from other disorders (15). We recently reported the MCP width as novel biomarker for FXTAS (16); decreased MCP width was observed in individuals who later developed symptoms of FXTAS as compared to premutation carriers (PM) who did not, and healthy controls. In addition, we also found reduced midbrain and area of the pons cross-sectional areas in patients with FXTAS compared to PM without FXTAS and controls (16). In a more recent study, we reported the association between these brain measures, including reduced MCP and SCP width, midbrain, and area of the pons cross-sectional area with increased expression levels of the *Iso10/10b, Iso4/4b FMR1* mRNA isoforms of the *ASFMR1* 131 bp mRNA isoform (17), suggesting their potential role in the pathogenesis of FXTAS.

Metabolic alterations and mitochondrial dysfunction have been extensively investigated in numerous age-related neurodegenerative disorders (18). However, the relationships between systemic abnormalities in metabolism and the pathogenesis of FXTAS are poorly understood. Previous metabolomic studies have investigated a panel of four core serum metabolites (phenethylamine (PEA), oleamide, aconitate, and isocitrate) for sensitive and specific diagnosis of the PM with and without FXTAS and found oleamide/isocitrate as a biomarker of FXTAS (19). Later, mitochondrial dysfunction, markers of neurodegeneration, and pro-inflammatory damage in PM were reported (20). Increased mitochondrial oxidative stress in primary fibroblasts derived from PM, compared with age and sex-matched controls has also been observed (21). Napoli and colleagues found the presence of the Warburg effect (which involves an increase in the rate of glucose uptake and preferential production of lactate, even in the presence of oxygen) in the peripheral blood mononuclear cells (PBMCs)'s derived from the controls, in PM with and without FXTAS (22). Later, Napoli et al. observed a significant impact of allopregnanolone treatment on oxidative stress, GABA metabolism, and mitochondria-related outcomes, and suggested allopregnanolone as a potential therapeutic for the cognitive and GABA metabolism improvement in FXTAS patients (23). In the premutation animal model's significant metabolic changes were found in the sphingolipid and purine metabolism in the cerebellum of premutation mice while the *Schlank (Cers5), Sk2 (Sphk1)*, and *Ras (Impdh1)* genes were suggested as genetic modifiers of CGG toxicity in Drosophila (24). It is, however, unclear how global perturbations in metabolism may be related to severity of FXTAS pathology and the eventual expression of symptoms in individuals at risk for developing FXTAS. Our recent study identified metabolic biomarkers of FXTAS early diagnosis and disease progression by characterizing individuals who developed symptoms of FXTAS over time. Specifically, we found that lipid metabolism and specifically the sub pathways involved in mitochondrial bioenergetics, are significantly altered in FXTAS (25).

To date, no study evaluating the metabolic alterations in correlation with brain changes in PM who develop symptoms of FXTAS over time has been reported. In the current study, we evaluated male participants, carriers of the *FMR1* premutation allele, enrolled in an ongoing longitudinal study carried out at the UC Davis MIND Institute. The participants were followed for at least two longitudinal time points (Visit 1, V1, and Visit 2, V2) during which neuroimaging, neuropsychological, molecular measurements, as well as medical and neurological examinations were collected. A subset of the premutation participants, all symptom-free at the time of enrollment, developed symptoms that warranted a diagnosis of FXTAS by Visit 2. We define these individuals as converters (CON). The remaining premutation participants, who did not develop symptoms of FXTAS by Visit 2, we define as non-converters (NCON). In the current work, we investigated whether the expression levels of identified metabolic biomarkers were associated with changes in brain measures including the midbrain and pons cross-sectional area and MCP width, in the CON group compared to the NCON and HC groups. In addition, we also investigated the association of metabolite expression with the progression of FXTAS. Understanding the metabolic variations along with brain changes in PM who developed FXTAS symptoms over time is likely to provide insights into novel disease-modifying treatments for this progressive neurodegenerative disorder.

## Materials and Methods

### Study Participants

As part of a continuing longitudinal study at UC Davis MIND Institute, male PM, >45 years of age, and non-carrier age-matched controls were recruited from throughout the USA and Canada [as detailed in (16)]. All male participants were white in race; there were three Hispanic participants in the HC group, one in the CON group, and zero in NCON group. The studies and all protocols were carried out in accordance with the Institutional Review Board at the University of California, Davis. All participants gave written informed consent before participating in the study in line with the Declaration of Helsinki. FXTAS stage scoring was based on the clinical descriptions as previously described (26). Three categories were used in the diagnosis of FXTAS as explained in Zafarullah and Tassone (27) and termed as “definite,” “probable” and “possible.” Three age-matched groups were included in this study: CON, NCON, and HC. Using the data from two brain scans, from neurological assessment, FXTAS stage, and CGG repeat length, 10 participants were classified as “CON” as they developed clear FXTAS symptomology between visits (FXTAS stage score was 0–1 at V1 and ≥2 at V2); 10 were defined as “NCON” because they continued to show no signs of FXTAS at V2 (FXTAS stage score was 0–1 at both V1 and V2) and 10 as HC (normal *FMR1* alleles/non-PM).

### CGG Repeat Length

Genomic DNA (gDNA) was isolated from 5 mL of peripheral blood leukocytes using the Gentra Puregene Blood Kit (Qiagen). CGG repeat allele size and methylation status were assessed by using the combination of Southern Blot and PCR analysis as previously reported (28, 29).

### Brain Measures

The following methods including MRI acquisition and MRPI analysis were originally described in our previous report (16). High resolution structural magnetic resonance imaging (MRIs) acquisition was obtained on a 3T Siemens Trio scanner using a 32-channel head coil and a T1-weighted 3D MPRAGE sequence with the following parameters: *TR* = 2,170 ms, *TE* = 4.86 ms, flip angle = 7°, FoV = 256 mm2, 192 slices, 1 mm slice thickness. The scans were first aligned along the anterior-posterior commissure line using acpc detect (http://www.nitrc.org/projects/art) (30) or manually using DTI Studio (www.mristudio.org) (31). Then MRI bias field correction was performed using N4 (http://stnava.github.io/ANTs/) (32). A series of independent raters (two per measure) who were blinded to the participant age, group, and time point, quantitatively assessed all MR images for four measurements of brain morphology: MCP width as well pons and midbrain cross-sectional areas were based on methods previously described (33, 34).

### Sample Preparation and Metabolite Profiling

Plasma metabolite profiling was determined by a non-targeted platform that allows the relative quantitative analysis of a large number of molecules (35). Samples were stored at −80°C until processing and then prepared using the automated MicroLab STAR® (Hamilton Company, Reno, NV, USA). Several recovery standards were added prior to the first step in the extraction process for QC purposes. To remove protein, dissociate small molecules bound to protein or trapped in the precipitated protein matrix, and to recover chemically diverse metabolites, proteins were precipitated with methanol under vigorous shaking for 2 min (Glen Mills GenoGrinder 2000) followed by centrifugation. The resulting extract was divided into five fractions: two for analysis by two separate reverse phases (RP)/UPLC-MS/MS methods with positive ion mode electrospray ionization (ESI), one for analysis by RP/UPLC-MS/MS with negative ion mode ESI, one for analysis by HILIC/UPLC-MS/MS with negative ion mode ESI, and one sample were reserved for backup. Samples were placed briefly on a TurboVap® (Zymark) to remove the organic solvent. The sample extracts were stored overnight under nitrogen before preparation for further analysis as explained in Zafarullah et al. (25).

### Statistical Analysis

The association between brain measures and metabolites at a single visit was analyzed using linear regression models that included a brain measure as the area of the pons and a metabolite as the single covariate. The association between changes in brain measures and in metabolites between visits was analyzed using linear regression models that included change in a brain measure as the area of the pons and change in metabolite, baseline metabolite level, and baseline brain measure as covariates. Models fitted to visit 1 data included all subjects (control, NCON, and CON), and models fitted to visit 2 data included all premutation subjects (NCON and CON). Specifically, all the Visit 1 regression analyses included all subjects (*n* = 30), and all the Visit 2 regression analyses included all premutation subjects (*n* = 20). *P*-values were adjusted for multiple testing (within each analysis, across metabolites) using the Benjamini-Hochberg false discovery rate controlling method (36). Analyses were conducted using R version 4.0.2 (37).

## Results

### Demographics

Three groups of male participants were included in this study: 1) PM who converted at V2 (CON; *n* = 10), 2) PM who did not convert at V2 (NCON; *n* = 10) and 3) healthy controls (HC; *n* = 10). All participants in the CON and NCOV groups were matched for age and CGG repeat length as reported in Table 1. Participant race, age, and ethnicity did not differ significantly between the three groups. As expected, CGG repeat size was significantly lower in healthy controls than in the CON and NCON groups (*P* < 0.001 in both comparisons) but it was not significantly different between the two premutation carrier groups of CON and NCON (*P* = 0.76).

**Table 1:** Demographic information on age and CGG repeats in three male participant groups: HC, CON and NCON.

		**Healthy controls (HC)**	**Converters (CON)**	**Non-converters (NCON)**	**All patients**	***P*-Value (*F*-Test)**
Age	N	10	10	10	30	0.936
	Mean (SD)	65.60 (3.239)	63.50 (6.786)	63.20 (4.849)	64.10 (5.101)	
	Median (Range)	64.50 (62–70)	63.50 (53–75)	64.00 (52–69)	64.00 (62–75)	
CGG	N	10	10	10	30	<0.001
	Mean (SD)	28.90 (4.095)	93.30 (22.91)	75.70 (18.73)	65.97 (32.26)	
	Median (Range)	30 (20–32)	84.50 (74–141)	74 (56–122)	72 (20–141)	

### Differential Metabolite Levels Linked With Area of the Pons Area in Healthy Control and Premutation Groups

**Figure 1:**
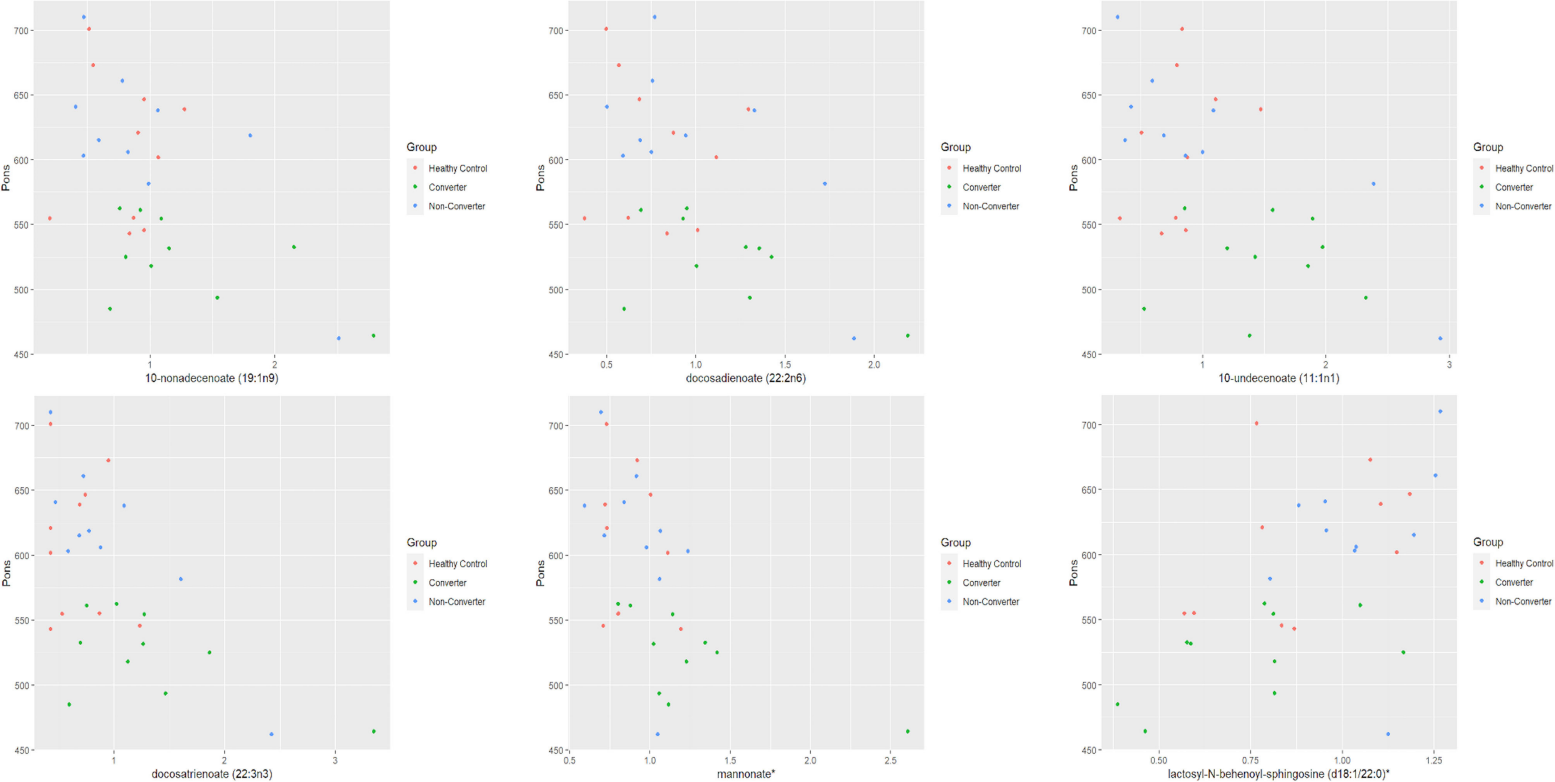
Pons by metabolite expression among HC, CON and NCON. Scatter plots showing correlation between pons and metabolite expression levels. The dots in red are representing the plotted values obtained for HC, green for CONV and blue for NCON. *The identity of these metabolites has not been officially confirmed based on the standard.

We have recently reported 94 potential metabolic biomarkers for early diagnosis and progression of FXTAS that showed significant changes in expression (*P* ≤ 0.05) in the CON as compared to the NCON both at V1 and V2 or only at V2 (25). In this study, we investigated the correlation between these potential metabolic biomarkers and brain measures (midbrain, area of the pons, and MCP width) among healthy control (HC), and PM including converter and non-converter (CON and NCON) at V1. We found a significant association (*P* ≤ 0.05) of expression level of six metabolites with area of the pons among all three groups (HC, CON, and NCON) at V1 (Figure 1). While no significant correlation of the midbrain and MCP width with the identified metabolites at baseline has been observed.

### Expression Levels of Metabolic Biomarkers Associated With Brain Measures

**Figure 2:**
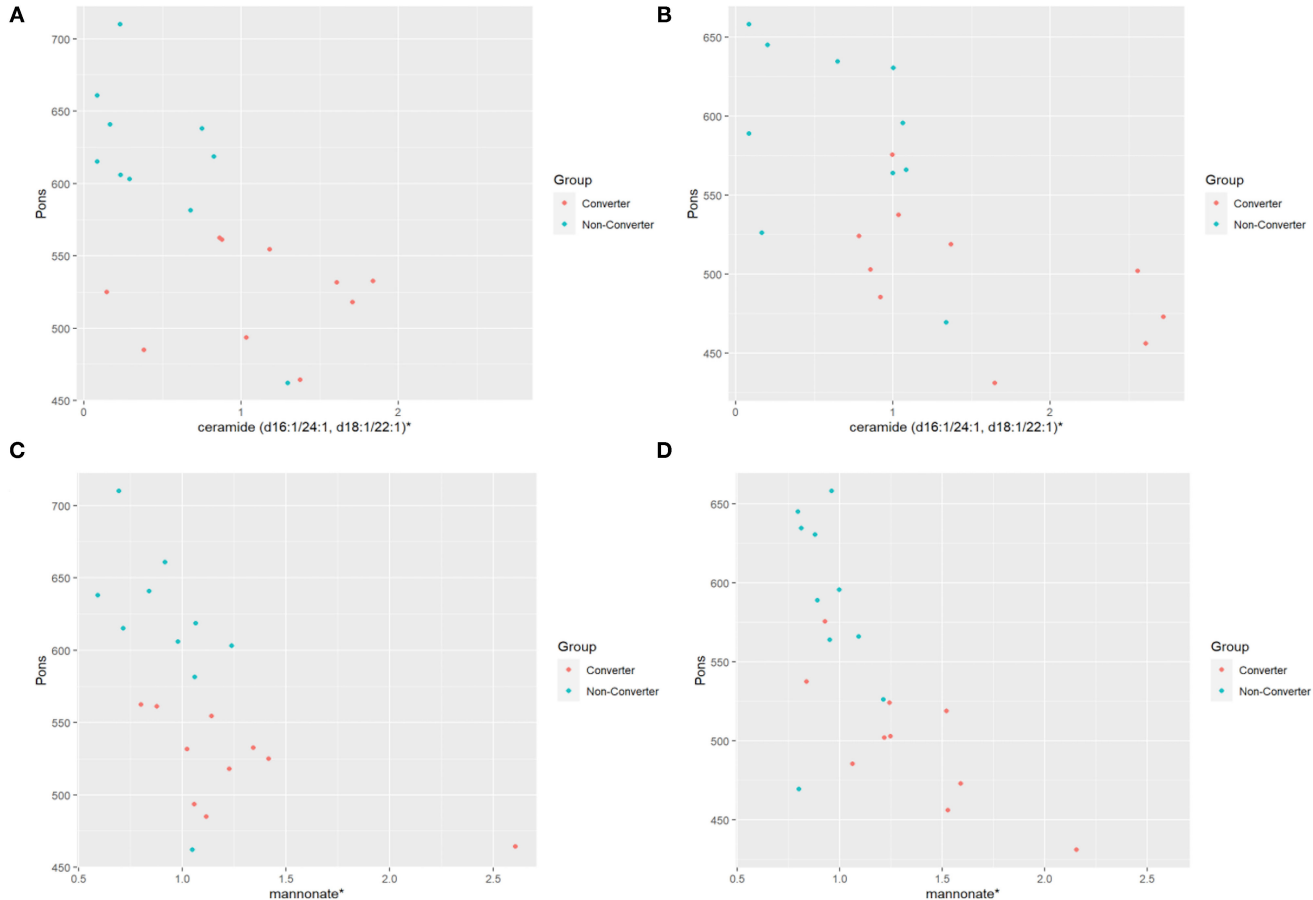
Distribution of metabolic biomarkers with pons between CON and NCON groups. **(A)** Scatter plots showing correlation between pons and ceramide in CON and NCON at V1. The dots in red are representing the plotted values obtained for CON, and turquoise for NCON. **(B)** Scatter plots showing correlation between pons and ceramide in CON and NCON at V2. **(C)** Scatter plots showing correlation between pons and mannonate in CON and NCON at V1. **(D)** Scatter plots showing correlation between pons and mannonate in CON and NCON at V2. *The identity of these metabolites has not been officially confirmed based on the standard.

Within the two premutation groups, the levels of 11 metabolites showed a significant correlation (*P* ≤ 0.05) with decreased area of the pons at V1 while four showed a significant correlation at V2 only in the CON group but not in the NCON group. Interestingly, level of ceramide (d16:1/24:1, d18:1/22:1) correlated with area of the pons area both at V1 [Regression Slope −72.3 (−118.7, −25.9); *P*-value 0.0496; Figure 2A] and V2 [Regression Slope −56.7 (−88.3, −25.2); *P*-value 0.0597; Figure 2B]. Similarly, we also observed a significant correlation between mannonate and area of the area of the pons both at V1 [Regression Slope −97.3 (−162.3, −32.3); *P*-value 0.0496; Figure 2C] and V2 [Regression Slope −135 (−203.8, −67.2); *P*-value 0.0543; Figure 2D]. No significant correlations were observed between the midbrain area and MCP width and any metabolites both at V1 and V2 between CON and NCON premutation groups.

### Metabolite Expression Levels Correlate With FXTAS Progression

**Figure 3:**
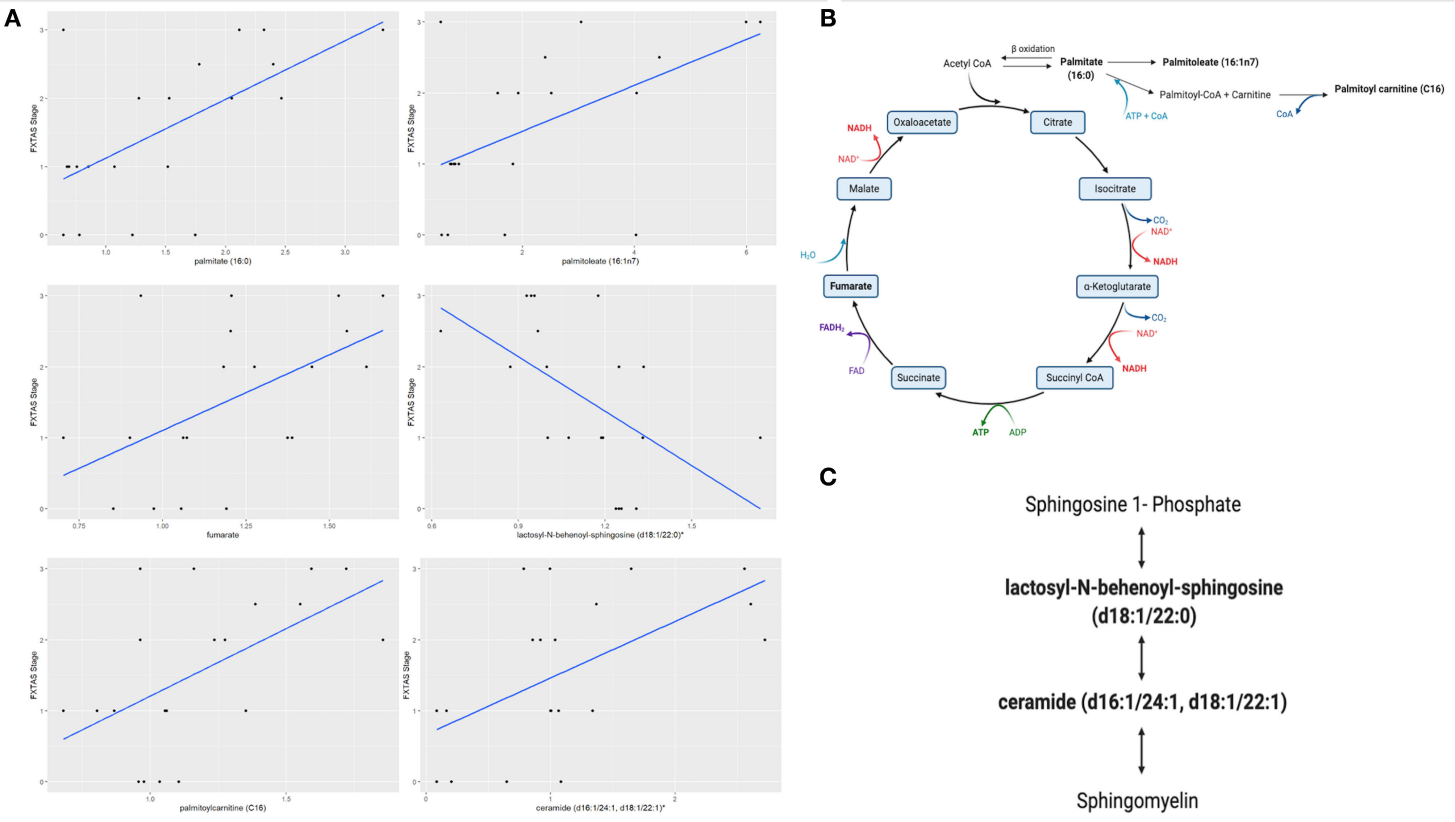
Fatty acid and sphingolipids metabolism associated with FXTAS development. **(A)** Six identified metabolites in FXTAS associated with other neurodegenerative disorder, here the comparison with the FXTAS stage is shown; data are presented as FXTAS stage (*y-*axis) and associated metabolite (*x*-axis) by using scatter plots. **(B)**. Disturbance of fatty acid and lipids metabolism pathway is shown. **(C)** Sphingolipid metabolism pathway is shown. Bold metabolites, *p* ≤ 0.05 linked with the development of FXTAS. *The identity of these metabolites has not been officially confirmed based on the standard.

We evaluated the differential expression of the metabolic biomarkers with the progression of FXTAS and with the FXTAS stage in the CON and NCON participants at V2. We observed that 27 metabolites significantly correlated with change in FXTAS stage from V1 to V2 with the majority of these metabolites being lipids followed by xenobiotics, amino acids, and energy (Table 2). Further we observed a significant correlation between the expression levels of several of these metabolites with the FXTAS stage (Figure 3A). Interestingly, six of these metabolites including palmitate (16:0) (Figure 3A), palmitoylcarnitine (C16), palmitoleate (16:1n7), fumarate, lactosyl-N-behenoyl-sphingosine (d18:1/22:0), and ceramide (d16:1/24:1, d18:1/22:1) have been reported to be critically involved in the development of other neurodegenerative disorders. In addition, these metabolites are part of the lipid and fatty acid metabolism (Figure 3B) and sphingolipid metabolism (Figure 3C). We previously shown that lipid metabolism was associated with the development and progression of FXTAS (changes of the FXTAS stage from V1 to V2) (25) and this association has also been reported in the premutation mouse model (24).

**Table 2:** Metabolite expression correlated with progression of FXTAS.

**Sr #**	**Super pathway**	**Metabolite**	**Regression slope (95% CI)**	**Raw *P*-value**	**Adjusted *P*-value**
1	Lipid	Myristate (14:0)	0.805 (0.376, 1.233)	<0.001	0.0541
2	Lipid	Pentadecanoate (15:0)	1.4 (0.569, 2.241)	0.00239	0.0541
3	Lipid	1-oleoylglycerol (18:1)	1.02 (0.399, 1.639)	0.00284	0.0541
4	Lipid	Margarate (17:0)	1.1 (0.429, 1.766)	0.00287	0.0541
5	Lipid	(14 or 15)-methylpalmitate (a17:0 or i17:0)	0.89 (0.347, 1.432)	0.00288	0.0541
6	Lipid	(2 or 3)-decenoate (10:1n7 or n8)	0.763 (0.273, 1.253)	0.00426	0.058
7	Lipid	Palmitate (16:0)	0.86 (0.30, 1.42)	0.00469	0.058
8	Lipid	10-heptadecenoate (17:1n7)	0.515 (0.175, 0.855)	0.00516	0.058
9	Xenobiotics	Mannonate^*^	1.87 (0.62, 3.12)	0.00566	0.058
10	Lipid	Behenate (22:0)^*^	0.817 (0.258, 1.376)	0.0066	0.058
11	Amino Acid	Trans-urocanate	−1.32 (−2.227, −0.404)	0.00716	0.058
12	Amino Acid	8-methoxykynurenate	1.36 (0.402, 2.313)	0.00796	0.058
13	Lipid	Ceramide (d16:1/24:1, d18:1/22:1)^*^	0.795 (0.232, 1.359)	0.00828	0.058
14	Xenobiotics	7-methylurate	−0.633 (−1.085, −0.181)	0.00868	0.058
15	Xenobiotics	3-bromo-5-chloro-2,6-dihydroxybenzoic acid^*^	0.847 (0.228, 1.467)	0.01013	0.058
16	Lipid	10-undecenoate (11:1n1)	0.995 (0.265, 1.725)	0.01035	0.058
17	Lipid	Ceramide (d18:1/17:0, d17:1/18:0)^*^	1.34 (0.35, 2.32)	0.01075	0.058
18	Lipid	Myristoleate (14:1n5)	0.392 (0.100, 0.684)	0.01127	0.058
19	Lipid	Palmitoylcarnitine (C16)	1.9 (0.469, 3.335)	0.01212	0.058
20	Lipid	Lactosyl-N-behenoyl-sphingosine (d18:1/22:0)^*^	−2.56 (−4.487, −0.624)	0.01235	0.058
21	Lipid	5-dodecenoate (12:1n7)	0.618 (0.139, 1.097)	0.0143	0.0595
22	Lipid	Palmitoleate (16:1n7)	0.323 (0.0722, 0.5745)	0.01451	0.0595
23	Lipid	N-behenoyl-sphingadienine (d18:2/22:0)^*^	1.69 (0.365, 3.010)	0.01525	0.0595
24	Lipid	Arachidate (20:0)	3.32 (0.71, 5.93)	0.01554	0.0595
25	Xenobiotics	3,7-dimethylurate	−0.652 (−1.167, −0.138)	0.01583	0.0595
26	Energy	Fumarate	2.13 (0.435, 3.828)	0.01663	0.0598
27	Lipid	3-hydroxymyristate	0.701 (0.14, 1.26)	0.01719	0.0598

* *The identity of these metabolites has not been officially confirmed based on the standard*.

## Discussion

The present study results provide evidence that brain measures, specifically the area of the pons cross-sectional area, correlate with plasma levels of metabolites that are part of the fatty acid and sphingolipid metabolism. These findings expand upon our previous study of plasma metabolic profiling of participants who developed symptoms of FXTAS over time (25), potentially representing biomarkers of early diagnosis and progression of FXTAS and suggest that these factors play a role in the brain structure of individuals with FXTAS.

Magnetic resonance imaging (MRI) takes advantage of a strong magnetic field for non-invasively imaging of parts of the brain parts to identify regional tissue abnormalities and to obtain volumes of brain structures. The imaging profile provides an opportunity to not only visualize the neuroanatomical and functional signatures of various neurodegenerative disorders, but it can also identify disease-specific biomarkers of the underlying processes. Various imaging biomarkers have been reported in Parkinson's disease (PD) (38), Amyotrophic Lateral Sclerosis (ALS) (39), Alzheimer Disease (AD) (40), and Dementia (41) and recently by our team in FXTAS (16).

The brainstem (which includes midbrain, area of the pons, and the medulla oblongata) is a critical regulator of vital bodily functions (42) with midbrain and area of the pons primarily supporting cognition and mood while medulla oblongata regulates cardiovascular and respiratory functions (43). Interestingly, lesions and atrophy of these brainstem structures represent the hallmarks of various neurological disorders and recent findings have pointed to a much deeper involvement of the brainstem nuclei which could change our understanding of the cause, prevalence and early diagnosis of these devastating diseases. Altered volume of midbrain, area of the pons, and medulla oblongata have been reported in individuals with schizophrenia (SCZ), bipolar disorder (BD), multiple sclerosis (MS), dementia, mild cognitive impairment (MCI), and Parkinson's disease (PD) in comparison to healthy controls (HC) (44, 45). Interestingly, reduction in area of the pons over time can significantly discriminate MSA from Progressive Supranuclear Palsy (PSP) (46). Moreover, the Fractional Anisotropy (FA) and Apparent Diffusion Coefficient (ADC) values in the area of the pons can differentiate the middle cerebellar peduncles parkinsonian subtype (MSA-P) patients from PD with 100% specificity (47). Interestingly, the voxel-based morphometry (VBM) analysis has also identified neurodegenerative changes primarily in the midbrain and area of the pons of PSP patients as compared to controls (48). Finally, degeneration of the locus coeruleus (LC), a long and narrow nucleus in the area of the pons, correlates with cognitive dysfunction and potentiate pathology of AD (49).

In our earlier studies, we observed the variation in the MCP width, area of the pons and midbrain cross-sectional areas as well as their significant association with the molecular measures in individuals who developed symptoms of FXTAS over time as compared to non-symptomatic PM and healthy controls, suggesting their role in FXTAS pathogenesis and progression (16, 17). These findings point toward the critical involvement of the area of the pons in neurodegenerative disorders, which could potentially provide information about the neuropathology of the disease and lead to early clinical diagnosis of these diseases.

Metabolomics is the omics platform that measures levels of metabolites in biological samples (50) uncovering potential biomarkers of aging and neurodegenerative diseases such as AD (51), Parkinson (52), Huntington (53), MS (54), and Amyotrophic Lateral Sclerosis (55) and FXTAS (25). A larger number of untargeted metabolomics-based studies have been reported using plasma/serum samples, due to its minimally invasive nature and relatively easy availability of blood samples. Unique metabolic signatures associated with altered energy homeostasis, Krebs cycle, changes in lipid membrane associated with abnormal CSF Aβ42 levels, altered mitochondrial function, neurotransmitter and lipid biosynthesis, are altered in plasma of patients with mild cognitive impairment and more pronounced in patients with AD (56–61). Majorly disturbed metabolic pathways observed in PD are also related to the metabolism of lipids, energy (TCA cycle, glycolysis, acylcarnitines), fatty acids and tryptophan, with the latter presenting a high correlation with the progression of PD (62–68). The energy and phospholipid metabolism have also been found to be impaired in patients with HD that ultimately affects the function of neurons (53, 69). Glucose metabolism is dysregulated in AD patients (70) and in area of the pons and cerebellum of MSA patients (71, 72), while an association of fatty acid metabolism with the development of ALS was observed (73). Finally, in our recent study we reported on the identification of metabolic biomarkers of early diagnosis and progression of FXTAS and on their association with altered lipid metabolism including free fatty acids, acylcarnitine, sphingolipids, diacylglycerol, and phospholipids, in individuals who developed the symptoms of FXTAS over time (25).

In this study we observed an association of metabolic biomarkers, including ceramides and mannonate, in CON as compared to NCON (Figure 2) with brain measures, specifically with area of the pons area, suggesting the potential role of altered metabolomics in the pathogenesis of FXTAS. We also found their significant association with the FXTAS stage (Table 2) ultimately providing the insight into the FXTAS disease progression with the dysregulation of the metabolic pathways.

The Krebs cycle or the TCA cycle is an important pathway in the production of ATP through the oxidative phosphorylation of acetyl-CoA in the mitochondria. With the onset of the neurodegenerative processes in PD, the metabolism of TCA cycle was found to be dysregulated indicating an energy shortage and mitochondrial dysfunction in PD (74). Similarly, previous studies in FXTAS (19, 20, 22) reported on altered plasma and PBMCs levels (either increased or decreased) of several intermediates of the Krebs cycle in individuals with FXTAS as compared to controls. In accordance with these previous studies, we found a significance correlation of various Krebs cycle intermediates, including palmitate (16:0), palmitoleate (16:1n7), palmitoylcarnitine (C16) and fumarate (Figure 3B, bold) with the FXTAS stage (Figure 3A) supporting the observed mitochondrial dysfunction as a contributing factor in the pathogenesis of the FXTAS.

Sphingolipids include ceramides, sphingosine-1-phosphate, lactosyl-N-behenoyl-sphingosine and sphingomyelins, which play an important role in neuronal functions as sphingolipids are critical to prevent the cell death, loss of synaptic plasticity, and neurodegeneration (75). High levels of ceramide have been detected in the CNS and in plasma of AD patients and of PD patients, indicating that ceramide metabolism could be associated with various stages of PD and AD progression and hippocampal atrophy (76–78) and suggested as a pharmacological target for the AD treatment (79). In a recent study, the sphingolipid metabolism, and specifically the levels of sphingosine, sphingosine 1-phosphate, and sphingomyelin were found to be altered in the cerebellum of FXTAS mice (24). We have reported on increased ceramides levels in the CON as compared to NCON group (25) and, interestingly, in this study we observed a significant association with area of the pons both at V1 and V2 (Figures 2A,B). Further, the sphingolipid metabolism intermediates lactosyl-N-behenoyl-sphingosine (d18:1/22:0) and ceramide (d16:1/24:1, d18:1/22:1) (Figure 3C, bold) both were significantly associated with FXTAS stage suggesting their role in the development of FXTAS and the pathway as a potential target for personalized therapeutic development.

## Conclusion

In this study, we found a significant correlation of metabolic biomarkers with the area of the pons in individuals who developed FXTAS over the time. We also report their significant association with the progression of the disorder and their role in context of dysregulated lipid and sphingolipid metabolism. These findings could be of a great value as the area of the pons provides distinct information about neuroanatomical and pathophysiological processes. Its association with the FXTAS biomarkers can assist in identifying the PM at risk as well as assist in evaluating disease progression and therapeutic responses to targeted drug development. Further research is needed to replicate these findings in a larger well-characterized cohort to further explore the role of other brainstem structures in FXTAS and human health and disease.

## Data Availability Statement

The raw data supporting the conclusions of this article will be made available by the authors, without undue reservation.

## Ethics Statement

This study and all the included protocols involved the human participant and were carried out in accordance with the Institutional Review Board at the University of California Davis. All participants gave written informed consent before participating in the study in line with the Declaration of Helsinki.

## Author Contributions

FT contributed to the conception while MZ, SR, DH, and FT contributed to the organization of the study. BD-J performed the statistical analysis and MZ wrote the first draft of the manuscript. MZ, BD-J, EF, SR, DH, and FT made amendments to the manuscripts. All authors contributed to the manuscript revision and approved the submitted version, recruited samples in their respective centers, assessed outpatients, and organized the database.

## Conflict of Interest

DH has received funding from Novartis, Roche, Seaside Therapeutics LLC and Marinus Pharmaceuticals, Inc. for designing Fragile X clinical Trials. FT has received funding from Azrieli Foundation, Zynerba and Asuragen, Inc. for studies in Fragile X syndrome. The remaining authors declare that the research was conducted in the absence of any commercial or financial relationships that could be construed as a potential conflict of interest.

## Publisher's Note

All claims expressed in this article are solely those of the authors and do not necessarily represent those of their affiliated organizations, or those of the publisher, the editors and the reviewers. Any product that may be evaluated in this article, or claim that may be made by its manufacturer, is not guaranteed or endorsed by the publisher.
